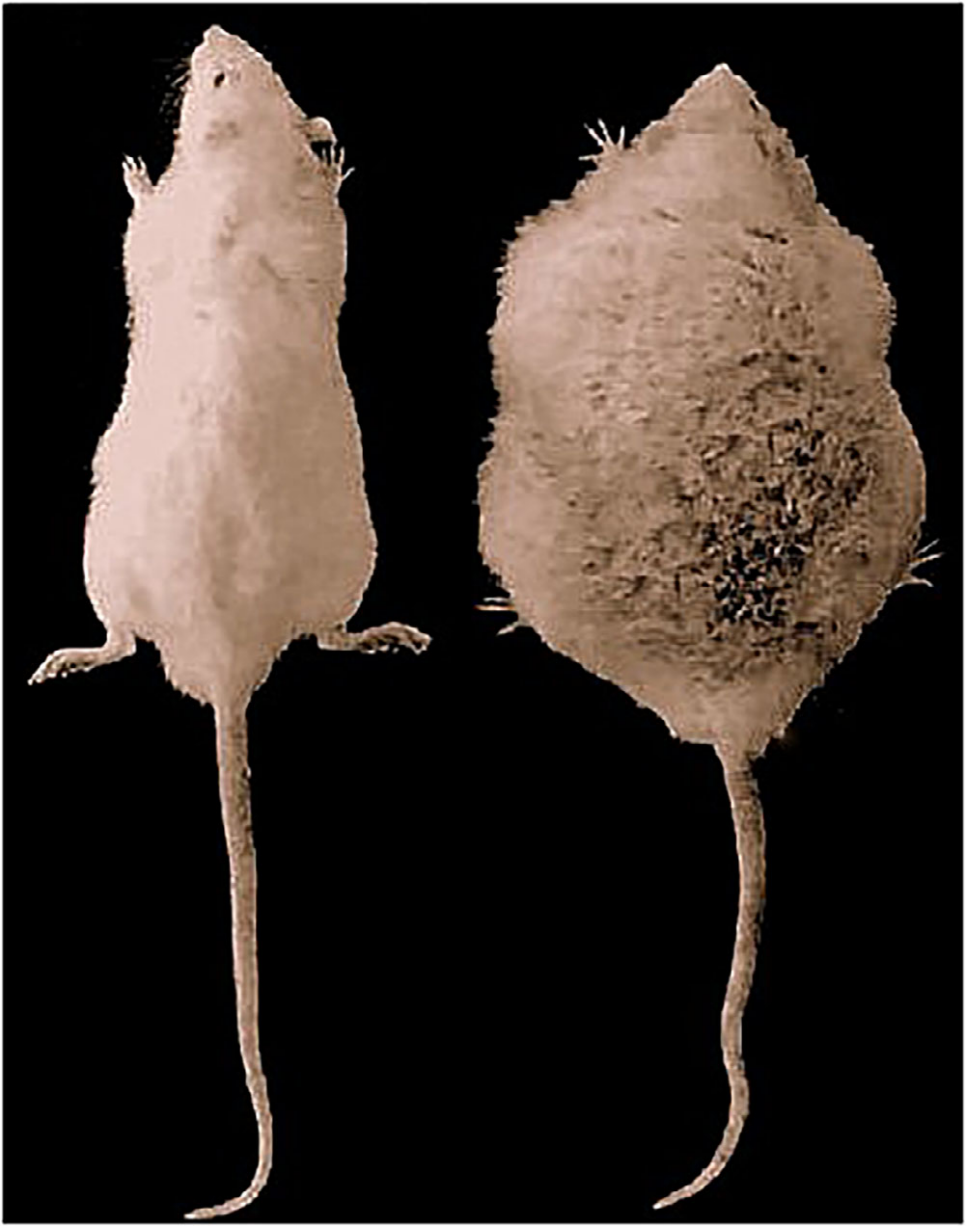# Interconnected Dynamics of ‘Obesageing’: Intricate Relationship Between Obesity and Ageing Processes

**DOI:** 10.1002/alz.085889

**Published:** 2025-01-03

**Authors:** Shampa Ghosh, Krishna Kumar Singh, Suchita Jha, Manchala Raghunath, Jitendra Kumar Sinha

**Affiliations:** ^1^ ICMR ‐ National Institute of Nutrition, Hyderabad, Telangana India; ^2^ GloNeuro Academy, Noida, Uttar Pradesh India; ^3^ Symbiosis Centre for Information Technology, Pune, Maharastra India; ^4^ Symbiosis Institute of International Business, Pune, Maharastra India

## Abstract

**Background:**

Obesity and ageing individually pose significant challenges to global public health, leading to preventable deaths. With an increasing geriatric population due to improved medical interventions, the intersection of these health issues becomes a critical concern worldwide. Both developed and developing countries grapple with the consequences of obesity, a major risk factor for various conditions like cardiovascular diseases, hypertension, type 2 diabetes, dementia, and neuropsychiatric diseases. Ageing, a natural process involving the gradual decline in physiological functions, shares similar comorbidities with obesity.

**Method:**

This paper explores the concept of ‘Obesageing,’ highlighting the commonalities between the pathological aspects of obesity and the normal physiological processes of ageing. A unique rodent model, WNIN/Ob, has been established to mimic Obesageing, embodying traits of morbid obesity and premature ageing.

**Result:**

The WNIN/Ob rodent model provides a platform to unravel the intricate interplay of mechanisms shared between obesity and ageing. This novel approach enhances our understanding of the complex relationship, paving the way for future strategies to address the escalating challenges posed by the simultaneous rise of obesity and ageing.

**Conclusion:**

The development of the WNIN/Ob rodent model signifies a breakthrough in comprehending the interconnected dynamics of obesity and ageing. This model serves as a valuable tool for investigating shared mechanisms, enabling the formulation of targeted strategies to mitigate the dual burden of obesity and ageing in the global population.